# Supplementary material for: Comprehensive Linear Epitope Prediction System for Host Specificity in Nodaviridae
Source: Viruses. 2022 Jun 22;14(7):1357. doi: 10.3390/v14071357 (PMC9319239; doi:10.3390/v14071357)
Supplement: Supplementary file 1 [file viruses-14-01357-s001.zip › viruses-1686420-supplementary.pdf]

Supplementary Table S1. Raw data of ELISA assay (all peptide samples were conducted by ELISA tests, and each sample was coated on 96 well microplate in triplicate). No. 1, 2, and 3 for pre-immunization triple experiments; 4, 5, 6 for after immunization triple experiments.

Peptides	1	2	3	Avg	Std	4	5	6	Avg	Std
**GNNV_CP$_{249\text{-}258}$**	0.182	0.175	0.213	0.2053333	0.01330415	0.352	0.264	0.295	0.5050	0.018318935
**BFNNV_CP$_{224\text{-}251}$**	0.22	0.211	0.265	0.232	0.028930952	0.481	0.415	0.422	0.439333	0.036253735
**BFNNV_CP$_{261\text{-}272}$**	0.194	0.244	0.24	0.226	0.027784888	0.395	0.388	0.554	0.445667	0.093884681
**BFNNV_CP$_{283\text{-}295}$**	0.19	0.209	0.215	0.2046667	0.013051181	0.423	0.357	0.441	0.4070	0.044226689
**BFNNV_CP$_{300\text{-}321}$**	0.241	0.231	0.239	0.2146667	0.01504438	0.511	0.485	0.503	0.4470	0.01637071
**DGNNV_CP$_{221\text{-}238}$**	0.189	0.23	0.203	0.2073333	0.020840665	0.352	0.53	0.408	0.4300	0.091016482

Supplementary Table S2. All experimental data were analyzed by multiple T test (Holm-Šídák method).

Peptide name	Significant differences	P value	After immunization (Mean value)	Pre-immunization (Mean value)	Difference	Standard error of difference	T ratio	degree of freedom
GNNV_CP$_{249\text{-}258}$	Yes	0.000021	0.5050	0.2053	0.2997	0.01307	22.93	4.000
BFNNV_CP$_{224\text{-}251}$	Yes	0.001499	0.4393	0.2320	0.2073	0.02678	7.742	4.000
BFNNV_CP$_{261\text{-}272}$	Yes	0.017751	0.4457	0.2260	0.2197	0.05653	3.886	4.000
BFNNV_CP$_{283\text{-}295}$	Yes	0.001608	0.4070	0.2047	0.2023	0.02662	7.600	4.000
BFNNV_CP$_{300\text{-}321}$	Yes	0.000055	0.4470	0.2147	0.2323	0.01284	18.10	4.000
DGNNV_CP$_{221\text{-}238}$	Yes	0.014488	0.4300	0.2073	0.2227	0.05391	4.130	4.000
